# Mutplot: An easy-to-use online tool for plotting complex mutation data with flexibility

**DOI:** 10.1371/journal.pone.0215838

**Published:** 2019-05-15

**Authors:** Weiwei Zhang, Cheng Wang, Xuan Zhang

**Affiliations:** Department of Pathology and Microbiology, University of Nebraska Medical Center, Omaha, Nebraska; UMR-S1134, INSERM, Université Paris Diderot, INTS, FRANCE

## Abstract

With the development of technology, an enormous amount of sequencing data is being generated rapidly. However, transforming this data into patient care is a critical challenge. There are two difficulties: how to integrate functional information into mutation interpretation and how to make the integration easy to apply. One solution is to visualize amino acid changes with protein structure and function in web app platform. There are multiple existing tools for plotting mutations, but the majority of them requires programming skills that are not common background for clinicians or researchers. Furthermore, the recurrent mutations are the focus and the recurrence cutoff varies. Yet, none of the current software offers customer-defined cutoff. Thus, we developed this user-friendly web-based tool, Mutplot (https://bioinformaticstools.shinyapps.io/lollipop/). Mutplot retrieves up-to-date domain information from the protein resource UniProt (https://www.uniprot.org/), integrates the submitted mutation information and produces lollipop diagrams with annotations and highlighted candidates. It offers flexible output options. For data that follows security standards, the app can also be hosted in web servers inside a firewall or computers without internet with Uniprot database stored on them. Altogether, Mutplot is an excellent tool for visualizing protein mutations, especially for clinicians or researchers without any bioinformatics background.

## Introduction

The development of sequencing technology has revolutionized cancer studies. After almost two decades of development, Next-Generation Sequencing (NGS) is fast and affordable. It has made precision medicine a clinical reality. NSG provides comprehensive big data to individualize therapies in clinical settings and expand research information. Though this technological advancement has created more opportunities for treatment and research, it has also created a problem of efficiently synthesizing and summarizing the resulting data because they are so large and detailed. Manually filtering big data increases the chance of errors and organizing it is time-consuming. Big data is also difficult to effectively present. Software circumvents all of these problems. Several tools are available for this purpose. However, most are designed for users with programming backgrounds. This excludes hospital and the majority of institution users who do not have such a training. Mutplot offers functions work in web browser and provides flexibility for easy customization. It was designed specifically for clinicians and researchers to use on their own. It translates abstract big data into visual results. In addition, Mutplot is an open source tool works in all platforms and can be easily integrated inside of firewall for security purpose.

We compared Mutplot with other six most popular tools for mutation plots, including MutationMapper [1], Lollipops [2], Muts-needle-plot [3], Pfam [4], Plot Protein [5], and trackViewer [6]. None of them meets all the requirements for non-technical users (details shown in Table 1). All of them, except for MutationMapper, use command-line user interface that requires programming training. Muts-needle-plot, Plot Protein, and TrackViewer require manual domain input. Lollipops is unable to distinguish mutations with similar sample frequencies or clustered mutations. Besides, manually entering the data is prone to human errors, and it does not have mutation highlight function. Pfam output JSON file that is not a publishing format. MutationMapper seems to be the best choice because it uses web-based user interface, but it has its own drawbacks. It only displays the highest recurrent mutations (amino acid alterations) and this would eliminate driver gene mutations with low frequency [7]. In fact, many driver genes occur at very low variant allele frequencies due to inter-tumor genetic heterogeneity. If multiple mutations occur in the same gene, the MutationMapper could easily neglect the lower occurrence mutations that are critical for advancing cancer research and personalized medicine. In addition, if two variants are located too closely in MutationMapper, the information from one of them will be overlapped. Another pitfall of MutationMapper is that the domain name would be automatically truncated in the case of limited space. These shortcomings make MutationMapper less ideal for NGS analysis.

**Table 1:** Comparison between Mutplot and other most popular tools for mutaiotn plots.

Tools	y-axis	domain input	user interface	plot formats option	open source
**MutationMapper**	sample frequency	link to database	graphical user interface	PDF,SVG	no
**Lollipops**	none	link to database	command line	PNG,SVG	yes
**Pfam**	none	manual	graphical user interface	online graph	no
**muts-needle-plot**	sample frequency	manual	command line	SVG	yes
**trackViewer**	manual edit	manual	command line in R	R Graphics	no
**Plot Protein**	none	manual	graphical user interface	online graph	no
**Mutplot**	sample frequency	link to database	graphical user interface	JPEG,PDF,PNG,SVG	yes

Y-axis indicates the y-axis options in the plot. Domain input indicates the domain information is provided manually or automatically retrieve from database. User interface indicates users use command line or GUI to plot. Plot formats option indicates the options for output plot format. Open source indicates if users have source code to customize the tools.

## Materials and methods

**Fig 1:**
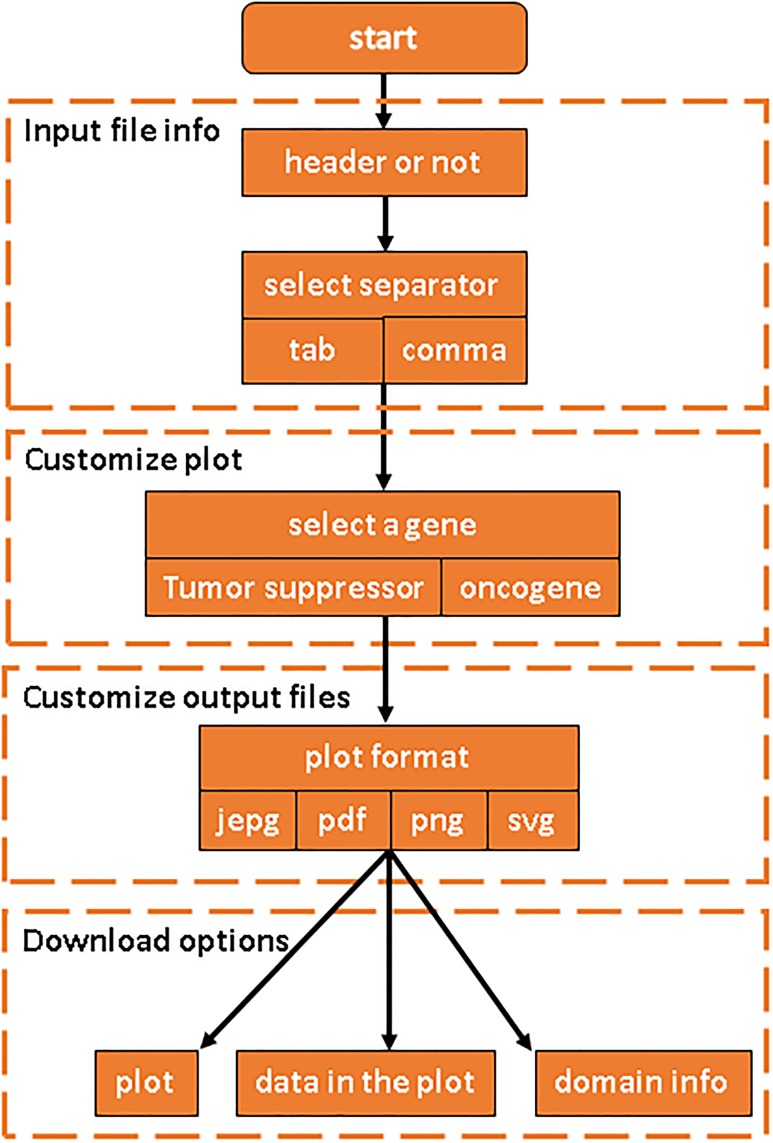
Mutplot workflow.

Mutplot includes a complete workflow for visualizing various protein mutations (Fig 1). After inputting a file (tab-delimited or comma-delimited format) with variants information (the required four columns are named Hugo_Symbol, Sample_ID, Protein_Change, and Mutaiton_Type, S1 Table), Mutplot automatically connects to the most updated protein information from the UniProt [8] database. A total number of 409 oncogenes and tumor suppressor genes are incorporated using a drop-down menu (S2 Table). Mutplot retrieves the domain information for the selected gene. The highlight options for amino acid frequency threshold are set as 1, 2, 3, 4, 5, 10, 15, 20, 25, 30. Both genes and highlight threshold options can be expanded by simply customizing the source code. The instruction is deposited in GitHub: https://github.com/VivianBailey/Mutplot.

Using the information, Mutplot generates protein diagrams with their domain information, amino acid position, mutation frequency, amino acid alteration, mutation type and the highlighted mutations. The amino acid positions are scaled to the gene length for accurate proportions. The highlighted mutation has detailed amino acid alteration information. Mutation type and description are color-coded for easy visualization and differentiation. When multiple mutations cluster together, Mutplot is smart enough to figure out how to label the mutation without interfering with other mutations. Mutplot also gives high flexibility in terms of output options. It supports JEPG, PDF, PNG as well as SVG for image download. It can also export the selected data for the diagram from the input data and the corresponding domain information retrieved from the updated Uniprot database.

The source code is available for non-commercial use in GitHub: https://github.com/VivianBailey/Mutplot and can be easily accessed, revised, or integrated in other pipelines or software. Revising the source code can shift Mutplot from a web app to a personal computer or server inside a firewall. This provides a great option for institutions that follow strict security regulations. In addition, the GitHub has a full documentation of Mutplot, instruction of how to customize the source code, and future releases are also deposited in the GitHub with description.

The web app was developed in R programming language. Packages shiny, ggplot2, plyr, httr, drawProteins and ggrepel are used.

## Results and discussion

**Fig 2:**
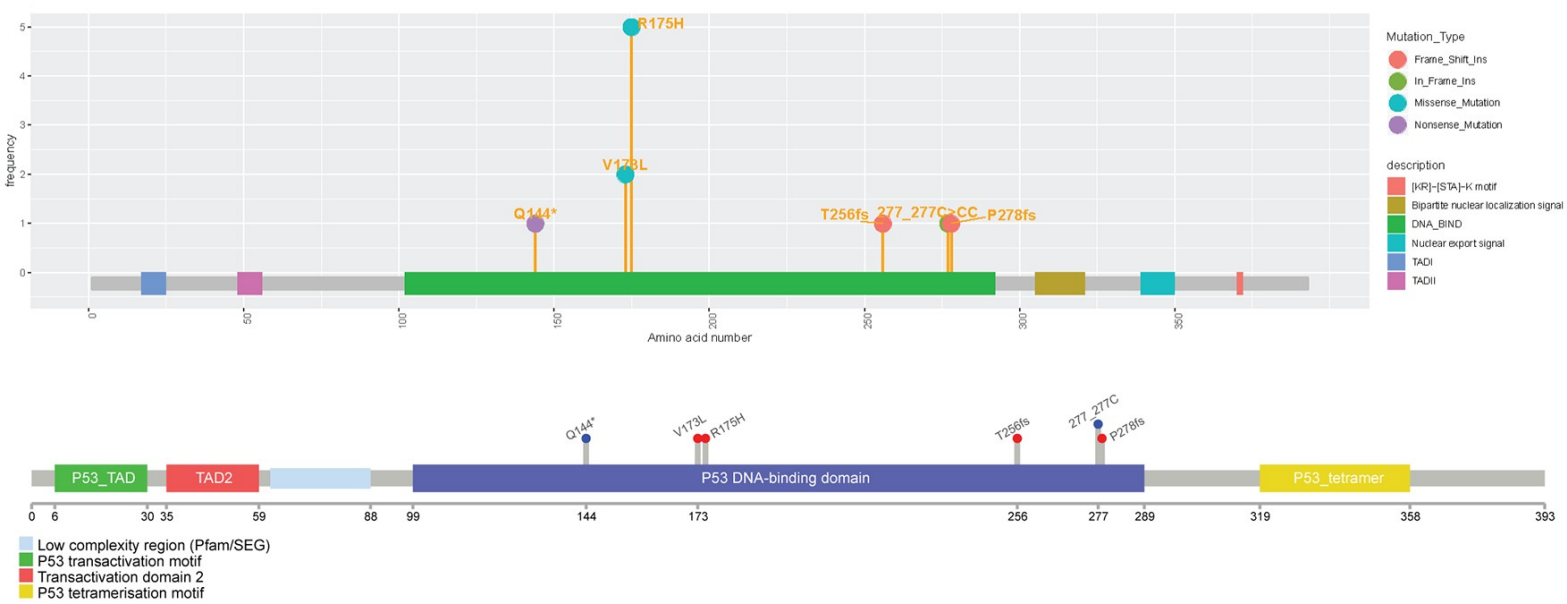
Comparison of single case *TP53* plots from Mutplot (top) and Lollipops (bottom). Same mutation settings applied to Mutplot and Lollipops. Mutplot has mutation types and better at repelling overlapping mutations.

We showed comparisons between Mutplot and Lollipops using the same example data. Fig 2 shows the same mutation settings in Lollipops and Mutplot. Lollipops was not designed for group patients analysis. Thus, it does not provide quantitative sample frequency information. Therefore, its ability to design target therapies based on recurrent mutations is limited. Mutplot is suitable for both single patient and group patients analyses. Mutplot also displays mutation types besides domain information and amino acid alterations. This provides important clues in regard to possible ways these mutations change protein functions. For example, missense mutation substitutes one amino acid in the protein, while nonsense mutation produces a truncated protein with transformed function or no function. In addition, Mutplot addresses the overlapping annotations issue by moving the labels. See the S1 File for details regarding lollipops and Mutplot comparison.

**Fig 3:**
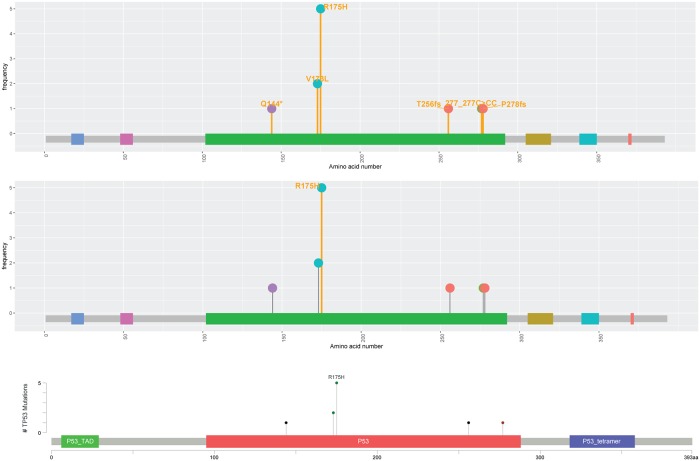
Comparison of *TP53* plots from Mutplot and MutationMapper. Mutplot can highlight and annotate mutations with any frequency. Mutplot can highlight both mutation frequency = 1 and mutation frequency >1 (top). Mutplot can highlight mutation frequency > = 5 (middle). MutationMapper only highlights the most frequent mutation (bottom).

**Fig 4:**
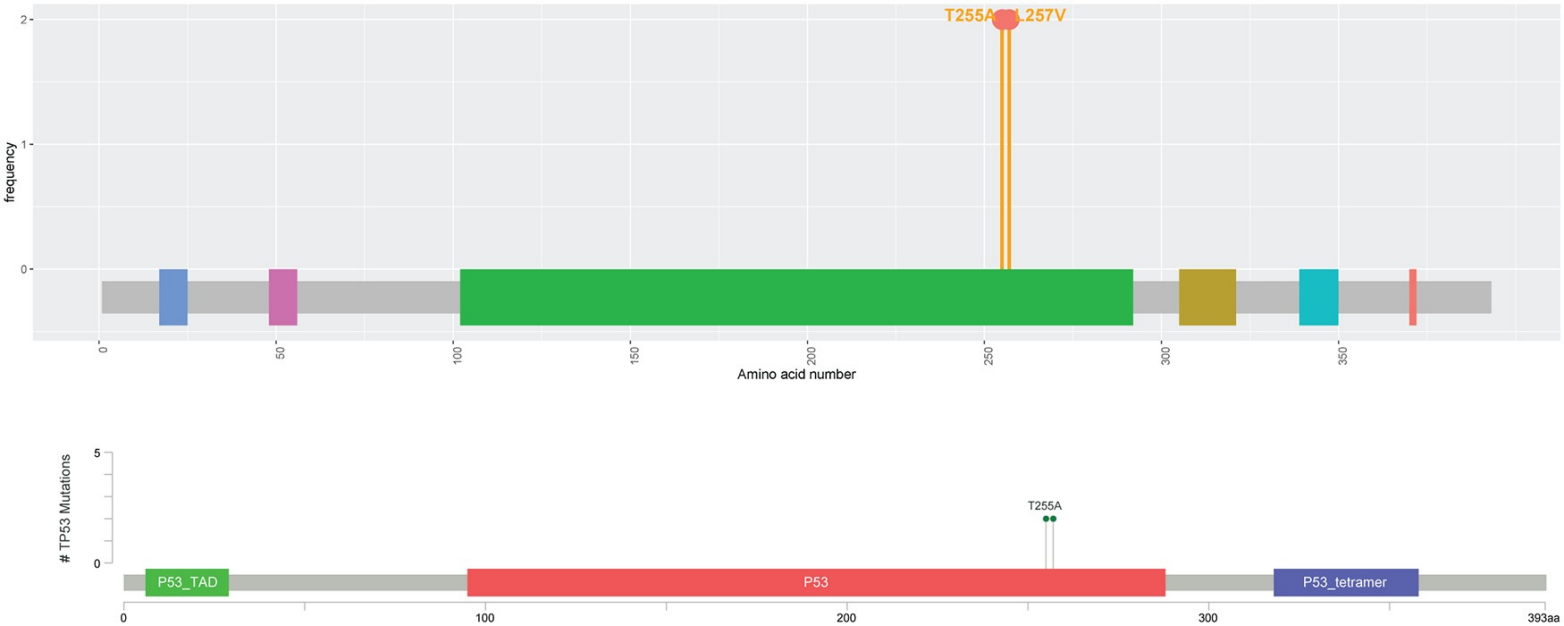
Comparison of overlapping labels from Mutplot and MutationMapper. When two mutations are next to each other, Mutplot is able to display both (Fig 4 top) and MutationMapper only displays one (Fig 4 bottom).

Fig 3 evaluates the MutationMapper and Mutplot using the same dataset. One improvement of Mutplot is the highlight flexibility through user-defined frequency cutoff. For example, when the frequency cutoff is set as 1, any mutation with a frequency equal to or higher than 1 will be highlighted (Fig 3 top). When the cutoff is set as 5, only mutations with a frequency equal to or higher than 5 will be highlighted (Fig 3 middle). In contrast, the MutationMapper only highlights the variants with the highest frequency (Fig 3 bottom). Besides, MutationMapper only annotates the most frequent variant. Though the other annotations could be displayed along with mouse movement, they stay hidden in the saved figures. In addition, Mutplot solves MutationMapper’s overlapping problem. When multiple variants locate at the same position, the MutationMapper lays one label over the others (Fig 4 bottom) which causes information loss. Mutplot adjusts the label positions when their mutations occur at the same location so that all labels can be displayed (Fig 4 top). Another drawback in MutationMapper that is fixed in Mutplot is domain name truncation when the space is limited. For example, *TP53* contains 3 main domains: *P53*_TAD, *P53*, and *P53*_tetramer. They are labeled as “*P53*…”, “*P53*” and “*P53*_tetr…” by MutationMapper (Fig 3 bottom and Fig 4 bottom), whereas Mutplot marks different domains by colors and lists their information in legends, which avoids the truncation.

## Conclusions

Big data is changing the scientific landscape dramatically. It brings significant cost advantages and faster and better approaches for decision-making. With the development of sequencing technology, we are getting such a huge amount of genome information but we don’t have the matching analysis power. More and more software and packages are available, but the majority of them are run by one or more programming languages. Scientists and physicians, who eventually need to draw conclusions or make decisions, have to rely on other bioinformatics. This is time-consuming for these decision makers, especially in precise medicine. Thus, easy-to-handle big data tools are in serious need.

Here, we present Mutplot, a web-based visualization tool for protein mutations. Mutplot retrieves protein data from the database automatically and builds diagrams displaying protein variants location, frequency etc. No programming skills are required. What’s more, Mutplot highlights the highly recurrent variants according to customer-defined cutoff. This function is especially useful when picking variants out of hundreds or even thousands of candidates in large cohort. In addition, Mutplot provides multiple publication-quality figure formats, such as PDF, JEPG, PNG, and SVG. Other outputs options including source data, protein domain information, are provided as well. For data under protection policy, Mutplot is also compatible with Linux web servers inside of firewall or computers without internet access. Source codes can be easily revised following the instructions on the program website at GitHub. This software simplifies data-processing, especially for medical researchers working with NGS.

## Supporting information

S1 Table An example of input file. (TXT)

S2 Table Oncogenes and tumor suppressor gene list. (TXT)

S1 File Lollipops and Mutplot comparison. (PDF)

## References

[1] Cerami E, Gao J, Dogrusoz U, Gross BE, Sumer SO, Aksoy BA, et al The cBio cancer genomics portal: an open platform for exploring multidimensional cancer genomics data. 10.1158/2159-8290.CD-12-0095 PMC3956037 22588877

[2] Jay JJ, Brouwer C. Lollipops in the clinic: information dense mutation plots for precision medicine. PloS one. 2016 8 4;11(8):e0160519 10.1371/journal.pone.0160519 27490490 PMC4973895

[3] Schroeder MP, Lopez-Bigas N. muts-needle-plot: Mutations Needle Plot v0. 8.0.

[4] Finn RD, Bateman A, Clements J, Coggill P, Eberhardt RY, Eddy SR, et al Pfam: the protein families database. Nucleic acids research. 2013 11 27;42(D1):D222–30. 24288371 10.1093/nar/gkt1223 PMC3965110

[5] Turner T. Plot protein: visualization of mutations. Journal of clinical bioinformatics. 2013 12;3(1):14 10.1186/2043-9113-3-14 23876180 PMC3724591

[6] Ou J, Wang YX, Zhu LJ, Ou MJ, GenomicAlignments I, GenomicFeatures G, et al. Package ‘trackViewer’.

[7] Gomez K, Miura S, Huuki LA, Spell BS, Townsend JP, Kumar S. Somatic evolutionary timings of driver mutations. BMC cancer. 2018 12;18(1):85 10.1186/s12885-017-3977-y 29347918 PMC5774140

[8] Apweiler R, Bairoch A, Wu CH, Barker WC, Boeckmann B, Ferro S, et al UniProt: the universal protein knowledgebase. Nucleic acids research. 2004 1 1;32(suppl_1):D115–9. 14681372 10.1093/nar/gkh131 PMC308865

