# Supplementary material for: Mutplot: An easy-to-use online tool for plotting complex mutation data with flexibility
Source: PLoS One. 2019 May 15;14(5):e0215838. doi: 10.1371/journal.pone.0215838 (PMC6519802; doi:10.1371/journal.pone.0215838)
Supplement: S1 File — (PDF) [file pone.0215838.s003.pdf]

Both lollipops and Mutplot provide the information of domain and amino acid position. In a single patient scenario, Mutplot and lollipops have exactly the same usage. But in a group patients scenario, lollipops may not present the mutation pattern clearly and would make it difficult to select genes for target panel. We compared lollipops with Mutplot in the group patients scenario where clinicians investigate mutation data to evaluate the sample frequency of mutated genes and gather select genes for target panel design, as shown below.

1. Evaluating sample frequency of mutated genes. Lollipops provide size specification for sample frequency. It works fine when the difference among sample frequencies is extreme and the mutations are far apart. However, the majority of sample frequencies are not extreme and mutations tend to cluster together in tumor cells. Users may find it difficult to distinguish the sample frequency difference using lollipops. For example, we applied the COUNT tag (lollipops -U P04637 R228Q@50 R248Q@100 R268Q@120 R278Q@150 R283Q@200 R285Q#00ff00@250 R288Q#7f3333@250) to scale the marker size in the first figure (the count number information marked in white). It is difficult to distinguish the count 100, 120, 150, 200, and 250 with bare eyes as the count 250 was labelled in both green and brown color, which somehow makes the brown dot looks smaller. Another example of four mutations cluster together in the second figure (lollipops -labels TP53 R278Q@150 R283Q@200 R285Q#00ff00@250 R288Q#7f3333@250), the text labels are overlapped and difficult to read. For large NGS data, four mutations clustered together is common.
2. Gathering information of selected genes for target panel development. When the investigation is complete, the next step is to document the used data for panel design. Mutplot can easily export the data including Uniprot domain information, mutation pattern figure with recurrence information, and subsetted uploaded original data. Lollipop does not have similar functions.

In summary, lollipops may not provide useful sample frequency information in a group patients scenario. And even in a single patient scenario, which lollipops is designed for, if mutations cluster together (only four mutations in the second example), lollipops cannot provide the same usage as Mutplot. In addition, lollipops may not quantify the difference through the plot as well as Mutplot performs (y-axis for sample frequency and grid and tick marks for assisting number reading). Mutplot provides data export option, summarizes UniprotID used in the clinical labs for oncogenes, provides options to change both UniprotID and gene lists for different purpose. As a result, Mutplot rarely need to request the information from bioinformaticians or manually input the UniprotID as lollipops does.

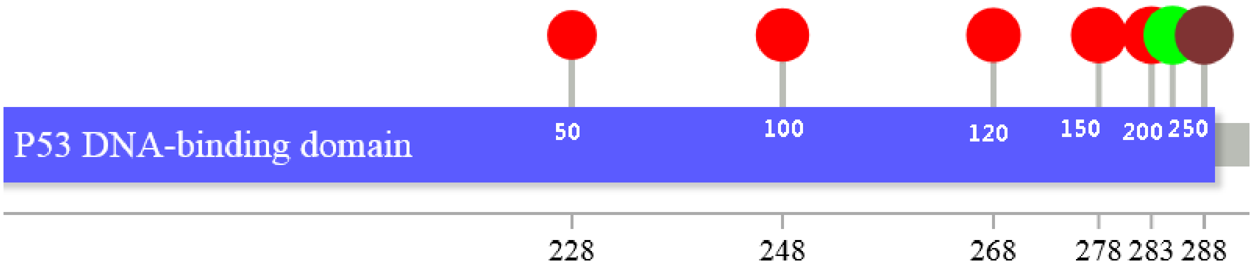

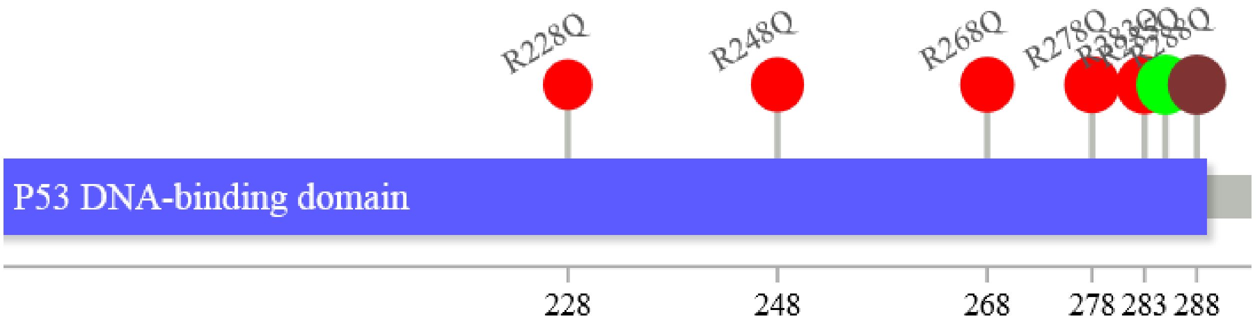

In addition, Mutplot is a better fit for clinical settings.
1. In the clinical setting, it is impractical to input 10 or more amino acid changes manually and prone to human errors.
2. Both Linux and Windows versions of lollipops require command line training. The lollipops itself is quite simple to use, the challenges are from the basic usage of command line environment
a. How to refer or add lollipops to the path before actually using it
b. Where to find the generated figures
c. How to generate the figures to a preferred directory
d. How to guarantee the users not to break down things in the command prompt
The first three challenges could be solved by training or providing simple templates but the fourth one is unpredictable, therefore, standard operating procedure cannot be written ahead of time. The lack of command line knowledge could bring errors in the process that should be avoided at all cost in clinical settings.

Overall, Mutplot has different usage in the scenarios describe above compared to lollipops. Mutplot works for both group patients scenario (MutationMapper is mainly designed for) and single patient scenarios (lollipops is mainly designed for) with user interface function and provides an option to run the program locally for security purpose.

Technical notes for testing lollipop: The most updated versions of lollipops are v1.5.0 and v1.5.1. They released on the same day. The difference between the two versions is the v1.5.1 fixed a crash with short data from uniprot and only has source code. According to the release notes (https://github.com/pbnjay/lollipops/releases), the two versions added the uniprot flag, the same database Mutplot uses. It seems like both versions were released without thorough testing and the author expects bug report from users to improve the program. We tested all the usage in the latest compiled lollipop v1.5.0 and explored the usages mentioned in early releases such as per-mutation color, use local file instead of Pfam API for graphic but was unable to find instruction in either the GitHub website or README.md comes with the program.
